# When Ultrasound and Hysterometry Disagree: Challenges in Levonorgestrel-Releasing Intrauterine System Placement

**DOI:** 10.7759/cureus.114261

**Published:** 2026-08-10

**Authors:** Ana Espirito Santo, Gonçalo Envia

**Affiliations:** 1 Family Medicine, USF Lapiás, ULS Amadora-Sintra, Amadora, PRT

**Keywords:** difficult intrauterine device insertion, hysterometry, intrauterine contraception, levonorgestrel-releasing intrauterine system, primary care, retroverted uterus, transvaginal ultrasound, uterine perforation

## Abstract

Insertion of a levonorgestrel-releasing intrauterine system (LNG-IUS) may be technically challenging in women with altered uterine anatomy or difficult cervical access. We report the case of a 24-year-old nulligravid woman with obesity and a subdermal contraceptive implant who sought transition to an LNG-IUS.

Pre-procedural transvaginal ultrasound (TVUS) demonstrated a retroverted uterus with apparently normal uterine dimensions. However, during insertion, hysterometry measured approximately 4 cm, raising concern regarding a reduced uterine cavity and possible perforation risk. The procedure was interrupted.

After cervical priming with misoprostol, following insertion was successfully performed. Follow-up TVUS confirmed correct positioning of the device near the uterine fundus.

This case highlights the potential discrepancy between anatomical uterine measurements obtained by ultrasound and functional assessment of the uterine cavity during instrumentation in retroflexed uteri and reinforces the importance of individualized contraceptive counseling and procedural caution in the primary care setting.

## Introduction

Long-acting reversible contraceptives (LARCs) are among the most effective contraceptive methods available and are increasingly used in primary care settings [1-4]. Recent evidence suggests that levonorgestrel-releasing intrauterine systems (LNG-IUSs) may improve dysmenorrhea severity, supporting their role as a therapeutic option beyond contraception [5,6]. LNG-IUSs offer highly effective contraception while also providing non-contraceptive benefits, including menstrual regulation and reduction of dysmenorrhea [1,3]. Although insertion is generally straightforward, uterine anatomical variations such as retroversion, retroflexion, cervical angulation, and apparent reduced uterine cavity length may complicate the procedure [7-9]. Difficult insertion may increase the risk of insertion failure, uterine perforation, malposition, procedural pain, and patient anxiety [7-9].

Assessment of uterine anatomy and uterine cavity length by transvaginal ultrasound (TVUS) and uterine sounding is commonly performed before insertion [1]. However, discrepancies between imaging findings and procedural hysterometry may occur, particularly in retroflexed uteri where alignment between the cervical canal and uterine cavity may be altered [10]. This report describes a clinically relevant case illustrating the importance of cautious procedural decision-making, reassessment strategies, and cervical preparation before repeat LNG-IUS insertion.

## Case presentation

A 24-year-old nulligravid woman with obesity (105 kg; BMI >35 kg/m²) attended a women’s health consultation in a primary care setting in 2025 (Day 0) seeking to transition from a 68-mg etonogestrel-releasing subdermal implant (Implanon NXT®, N.V. Organon, the Netherlands), inserted in 2023, to a 52-mg LNG-IUS (Levosert®, Gedeon Richter Plc, Hungary). Although the patient tolerated the implant well, she expressed preference for a long-acting intrauterine contraceptive method with the expectation of improving dysmenorrhea and menstrual-related symptoms.

**Figure 1:**
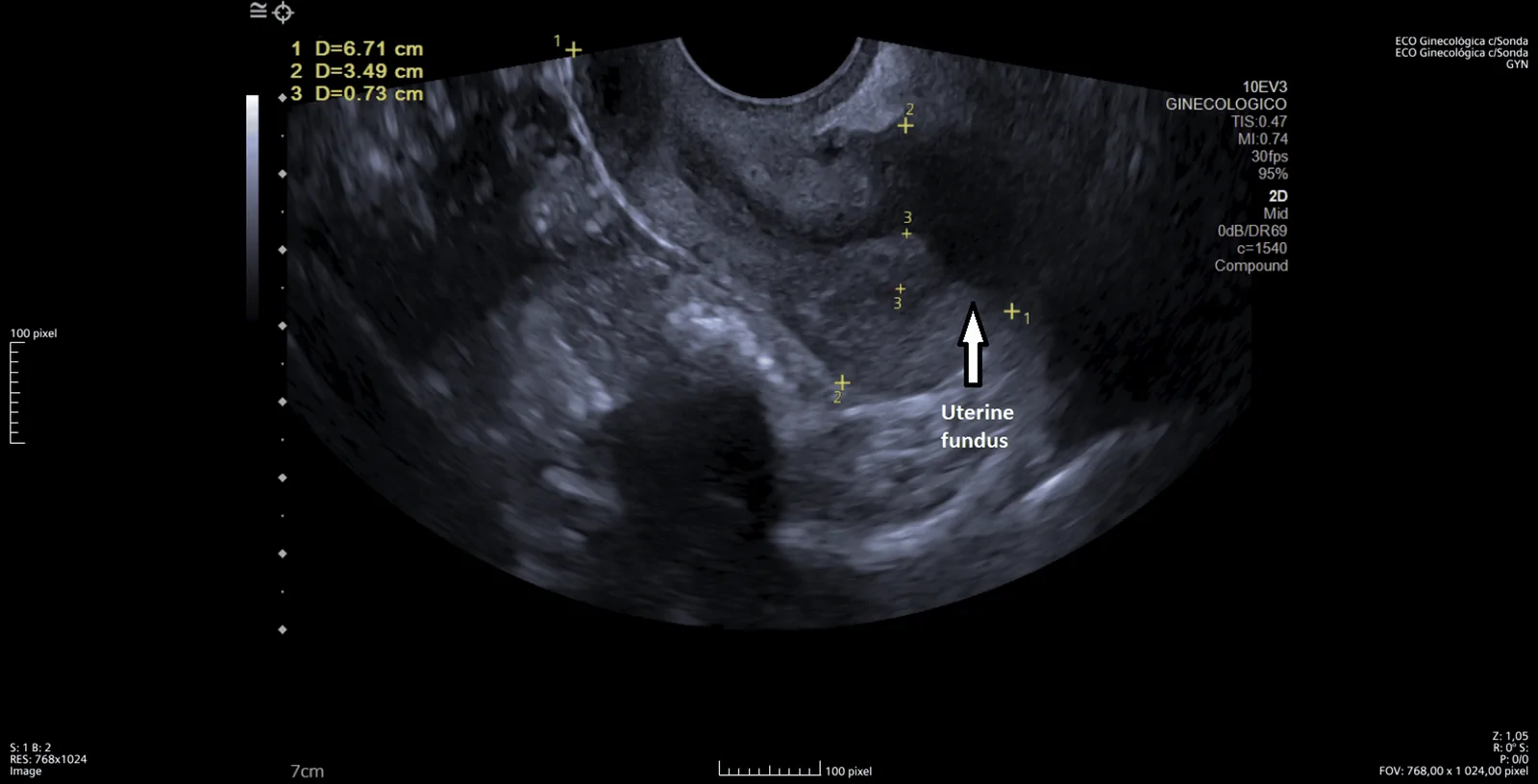
Initial TVUS demonstrating a retroflexed uterus with apparently normal uterine longitudinal dimensions (67 mm) prior to LNG-IUS insertion. The white arrow indicates the uterine fundus. TVUS: Transvaginal ultrasound; LNG-IUS: Levonorgestrel-releasing intrauterine system

Initial TVUS performed on Day 0 demonstrated a retroverted uterus with normal morphology and dimensions (67 mm longitudinally, 34 mm anteroposteriorly, and 37 mm transversely). The endometrium measured 7 mm and demonstrated a secretory pattern. Bilateral enlarged ovaries with multiple peripheral follicles compatible with polycystic ovarian morphology were observed. No adnexal masses or free pelvic fluid were identified (Figure 1).

During the first LNG-IUS insertion attempt, uterine sounding measured approximately 4 cm, substantially shorter than expected based on the pre-procedural ultrasonographic findings. Unexpected resistance was encountered during advancement of the uterine sound, precluding safe advancement beyond approximately 4 cm. Given the marked discrepancy between the ultrasonographic assessment and uterine sounding, together with concern regarding uterine perforation and uncertainty about the true uterine cavity trajectory, the procedure was prudently interrupted without forceful instrumentation or device placement [7,9].

**Figure 2:**
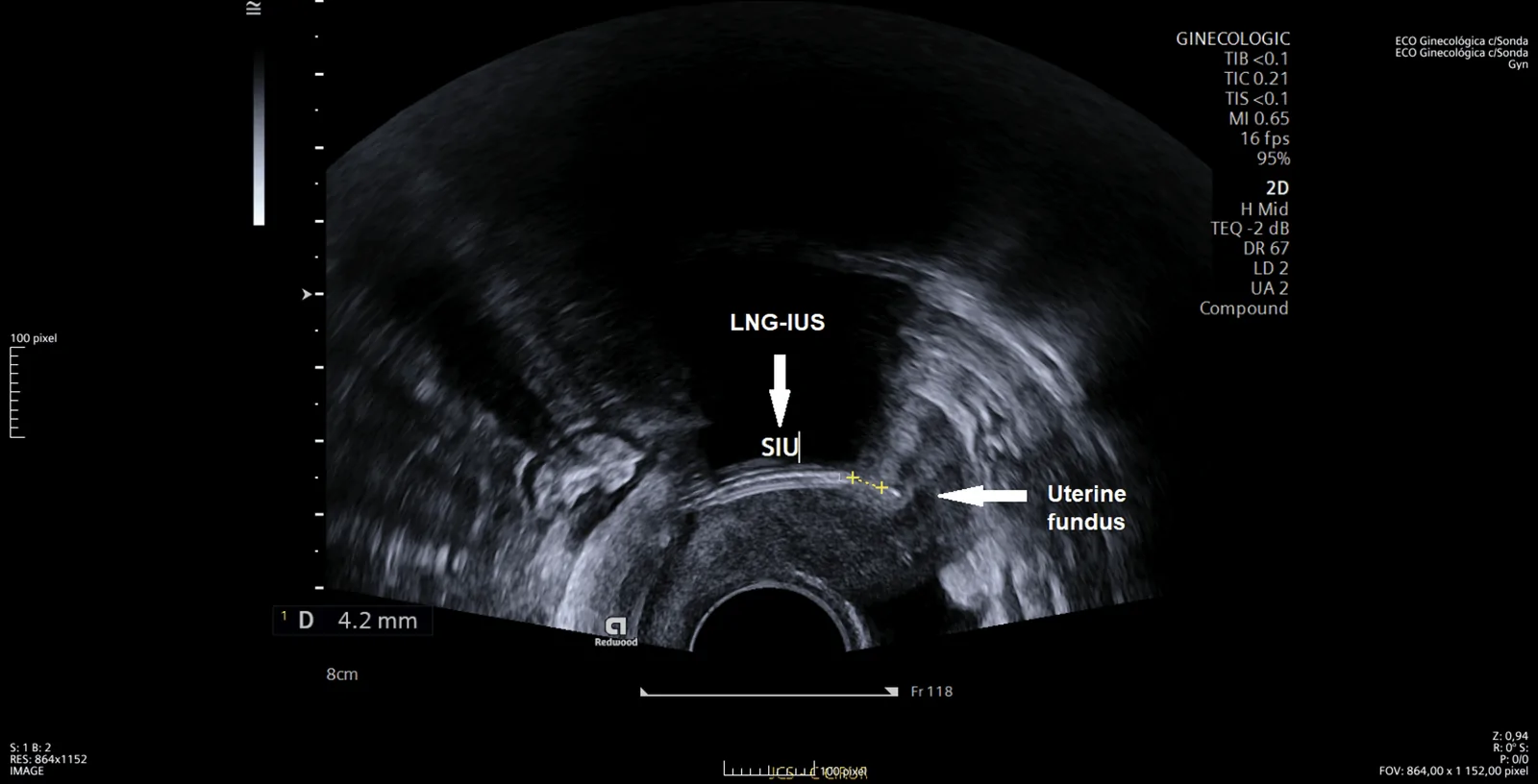
Follow-up TVUS (sagittal view) demonstrating correct fundal positioning of the LNG-IUS within a retroflexed uterus following the successful repeat insertion. The white arrows indicate the LNG-IUS (left) and the uterine fundus (right). TVUS: Transvaginal ultrasound; LNG-IUS: Levonorgestrel-releasing intrauterine system

**Figure 3:**
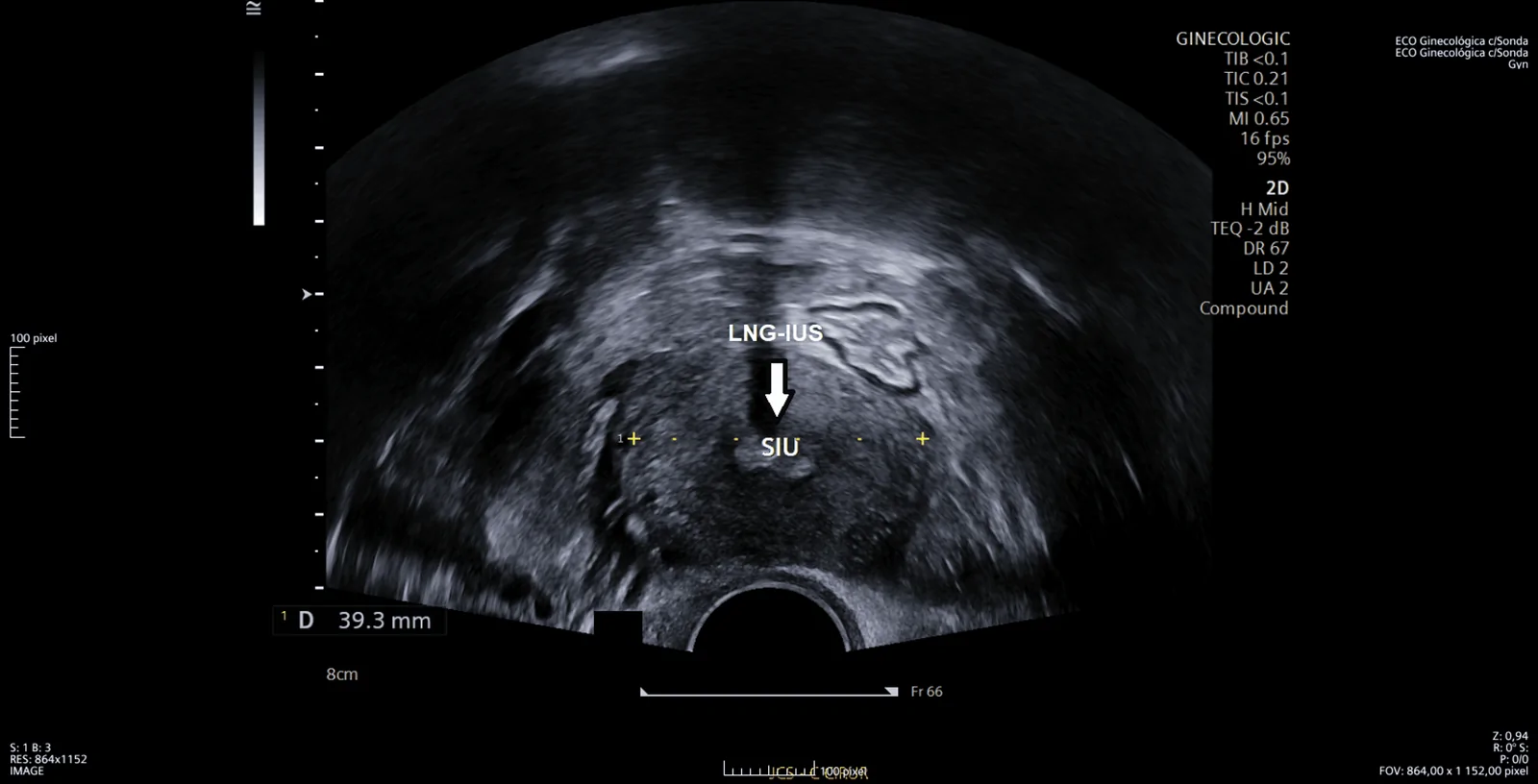
Follow-up TVUS (transverse view) confirming correct fundal positioning of the LNG-IUS. The white arrow indicates the upper end of the LNG-IUS near the uterine fundus. TVUS: Transvaginal ultrasound; LNG-IUS: Levonorgestrel-releasing intrauterine system

Cervical priming with oral misoprostol 200 mcg was prescribed and administered approximately 10 hours and four hours before the second insertion attempt. During Week 1, the repeat insertion was successfully completed without complications. Follow-up TVUS performed during Week 2 confirmed correct positioning of the LNG-IUS near the uterine fundus, with no evidence of perforation or other pelvic pathology (Figures 2, 3). The patient remained clinically stable and asymptomatic throughout follow-up.

## Discussion

Difficult LNG-IUS insertion remains clinically relevant, particularly in nulligravid women and in patients with obesity or altered uterine anatomy [1,3,4,7]. Retroverted or retroflexed uteri may impair alignment between the cervical canal and uterine cavity, increasing procedural difficulty and potentially leading to underestimation of functional cavity length during hysterometry [10].

Current European guidelines emphasize the importance of avoiding forceful insertion attempts when resistance or uncertainty regarding uterine depth is encountered [1]. In the present case, interruption of the first insertion attempt represented an appropriate safety-oriented clinical decision, particularly given concern regarding uterine perforation risk [7,9].

Notably, post-procedural ultrasound findings were more consistent with the initial ultrasonographic assessment than with the hysterometry measurement obtained during the first insertion attempt, suggesting that cervical-uterine angulation associated with uterine retroflexion, rather than a truly shortened uterine cavity, may have contributed to the unexpectedly short 4-cm hysterometry measurement.

The contribution of misoprostol to the successful repeat procedure cannot be determined from a single case; however, cervical preparation may have facilitated safer cervical instrumentation after the initial failed attempt.

Although bilateral polycystic ovarian morphology was identified on ultrasound, this finding was considered incidental and unrelated to the anatomical factors contributing to the difficult LNG-IUS insertion.

Evidence regarding routine cervical priming with misoprostol before intrauterine device (IUD) insertion remains heterogeneous; however, selective use after a failed insertion attempt may improve procedural success in difficult cases [11].

A recent Cochrane review evaluating misoprostol for IUD placement concluded that routine administration before standard insertion probably provides limited benefit regarding pain reduction or insertion success. However, women with a previous failed insertion attempt may experience clinically meaningful improvement in successful placement rates following misoprostol administration [12]. In the present case, selective cervical preparation after an interrupted first attempt may have contributed to successful insertion while avoiding forceful instrumentation in a retroflexed uterus [6,12].

Additional literature has emphasized the importance of cautious management during difficult IUD insertions, particularly in retroverted or retroflexed uteri. Cervical-uterine angulation may contribute to difficult sounding, failed insertion attempts, and increased perforation risk, reinforcing the need for careful reassessment rather than forceful instrumentation [10].

From a family medicine perspective, this report reinforces the importance of individualized contraceptive counseling, continuity of care, procedural caution, and longitudinal follow-up in community gynecology settings. Careful reassessment and shared decision-making allowed successful LNG-IUS placement while maintaining patient safety and respecting patient contraceptive preferences.

## Conclusions

This case highlights that retroverted or retroflexed uteri may complicate uterine sounding and LNG-IUS insertion, particularly when cervical-uterine angulation limits safe advancement of the uterine sound. An unexpectedly short hysterometry measurement in this setting may reflect the effects of cervical-uterine angulation rather than a truly shortened uterine cavity, warranting careful correlation with pre-procedural TVUS findings. When procedural findings are inconsistent with pre-procedural assessment, careful reassessment rather than forceful instrumentation may help minimize the risk of uterine perforation. In selected patients with previous failed insertion attempts or anatomically challenging uteri, selective cervical priming with misoprostol may facilitate successful repeat LNG-IUS insertion, although its contribution cannot be determined from a single case. This case also illustrates that challenging contraceptive procedures can be safely managed in primary care through careful clinical assessment, procedural caution, and individualized patient-centered care.
